# Synthesis, crystal structure and thermal properties of tetra­kis­(3-methyl­pyridine-κ*N*)bis­(thio­cyanato-κ*N*)nickel(II)

**DOI:** 10.1107/S2056989022011379

**Published:** 2023-01-01

**Authors:** Christian Näther, Inke Jess, Christoph Krebs

**Affiliations:** a Institut für Anorganische Chemie, Universität Kiel, Max-Eyth.-Str. 2, 24118 Kiel, Germany; Universität Greifswald, Germany

**Keywords:** synthesis, crystal structure, IR spectra, thermal properties

## Abstract

Reaction of Ni(NCS)_2_ with 3-methyl­pyridine in water leads to the formation of crystals of the title compound, [Ni(NCS)_2_(C_6_H_7_N)_4_]. All of them are of poor quality and non-merohedrally twinned but a refinement using data in HKLF-5 format leads to a reasonable structure model and reliability factors. The crystal structure of the title compound consists of discrete complexes, in which the nickel cations are sixfold coordinated by two terminal N-bonded thio­cyanate anions and four 3-methyl­pyridine ligands within slightly distorted octa­hedra. One of the 3-methyl­pyridine ligands is disordered and was refined using a split model. The discrete complexes are arranged into layers. X-ray powder diffraction proves that pure samples have been obtained, and in the IR spectrum, the CN stretching vibration is observed at 2072 cm^−1^, in agreement with the presence of only terminally coordinated thio­cyanate anions. Comparing the calculated powder pattern with those of the residues obtained by solvent removal from several solvates already reported in the literature proves that, in each case, this crystalline phase is formed. Assessing the crystal structures of the solvates in comparison with that of the ansolvate reveals some similarities.

## Abstract 2

In the crystal structure of the title compound, the nickel cations are octa­hedrally coordinated by two terminal N-bonded thio­cyanate anions and four 3-methyl­pyridine ligands.

## 1. Chemical context

Thio­cyanate anions are versatile ligands that can coordinate in many different ways to metal cations. The most common coordination is the terminal mode, in which these anionic ligands are only connected *via* the N or S atom, while the latter is only rarely observed. For several reasons, the μ-1,3 bridging coordination is more inter­esting and can lead to the formation of chains or layers (Näther *et al.*, 2013 ▸). There are also a few compounds with more condensed thio­cyanate networks that can form if these anionic ligands take up, for example, the μ-1,3,3 (N,S,S) bridging mode (Näther *et al.*, 2013 ▸).

We have been inter­ested in this class of compounds for several years targeting, for example, compounds that show inter­esting magnetic properties (Suckert *et al.*, 2016 ▸; Werner *et al.*, 2014 ▸, 2015*a* ▸,*b* ▸). In most cases, the neutral coligands used by us and others comprise pyridine derivatives and many such compounds have been reported in the literature (Mautner *et al.*, 2018 ▸; Böhme *et al.*, 2020 ▸; Rams *et al.*, 2020 ▸). If less chalcophilic metal cations such as Mn^II^, Fe^II^, Co^II^ or Ni^II^ are used, compounds with the composition *M*(NCS)_2_(*L*)_4_ (*M* = Mn, Fe, Co, Ni and *L* = pyridine derivative) are frequently obtained, in which the metal cations are octa­hedrally coordinated by two terminal N-bonded thio­cyanate anions and four coligands. Many of them have already been reported in the literature. If such compounds are heated, in several cases two of the coligands are removed, leading to a transformation to coligand-deficient compounds, in which the metal cations are linked by the anionic ligands and this is the reason why we are also inter­ested in such discrete complexes (Näther *et al.*, 2013 ▸).

**Figure 1:**
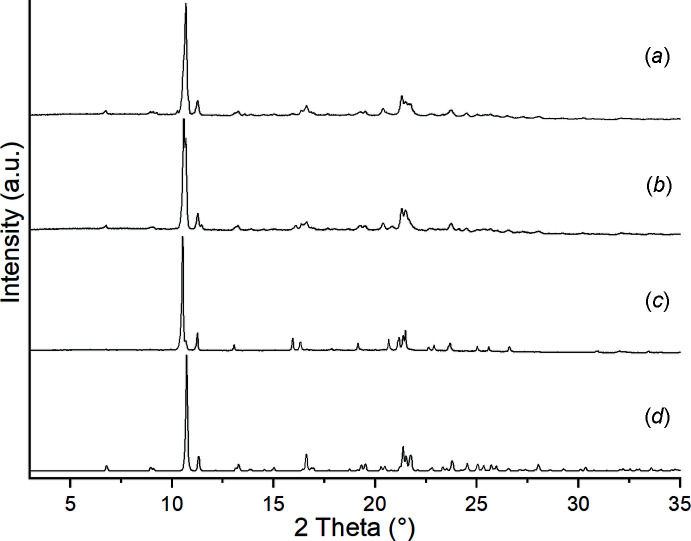
Experimental X-ray powder patterns of (*a*) [Ni(NCS)_2_(3-methyl­pyridine)_4_]·2aceto­nitrile, (*b*) [Ni(NCS)_2_(3-methyl­pyridine)_4_]·2ethanol, (*c*) [Ni(NCS)_2_(3-methyl­pyridine)_4_]·diethyl ether, each after 2 h in air, and (*d*) the calculated pattern of the title compound.

Throughout these investigations, we became inter­ested in Ni compounds with 3-methyl­pyridine as coligand for which some complexes have already been reported in the literature. However, all of these compounds consist of octa­hedral discrete complexes and the majority forms solvates with the composition Ni(NCS)_2_(3-methyl­pyridine)_4_·*X* with *X* = bis­(tri­chloro­methane) (LAYLOM; Pang *et al.*, 1992 ▸), which crystallizes in space group *P* 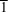 , bis­(di­chloro­methane) (LAYLIG; Pang *et al.*, 1992 ▸), which crystallizes in space group *C*2/*c*, mono-tetra­chloro­methane, mono-di­bromo-di­chloro­methane, mono-di­chloro­methane and mono-2,2-di­chloro­propane clathrates (JICMIR, LAYLAY, LAYLUS and LAYLEC; Pang *et al.*, 1990 ▸, 1992 ▸) as well as mono-tri­chloro­methane (CIVJEW and CIFJEW01; Nassimbeni *et al.*, 1984 ▸, 1986 ▸), all of which crystallize in the ortho­rhom­bic space group *Fddd*. Surprisingly, for unknown reasons, the crystal structure of the ansolvate is unknown. What is common to all of the solvates mentioned above is the fact that they contain non-polar solvents, which cannot coordinate to metal cations. We used solvents with donor atoms able to coordinate when attempting to prepare compounds with the composition Ni(NCS)_2_(3-methyl­pyridine)_2_(solvent)_2_. Upon heating, these should lose their two solvent mol­ecules, transforming into compounds with a bridging coordination. Surprisingly, even in this case, octa­hedral complexes with the composition Ni(NCS)_2_(3-methyl­pyridine)_4_·*X* (*X* = aceto­nitrile, ethanol, diethyl ether) were obtained (Krebs *et al.*, 2022 ▸). We have found that these solvates are unstable and have lost their solvents already at room temperature. X-ray powder diffraction (XRPD) proves that, independent of the crystal structure of the precursor, the same crystalline phase is always obtained (Fig. 1 ▸) which, according to IR spectroscopic data, bears only terminal N-bonded anionic ligands. Unfortunately no single crystals were obtained by this procedure, which means that the crystal structure of the ansolvate remained unknown. Starting from these observations, we tried to prepare crystals of the ansolvate using a variety of solvents and we eventually obtained crystals with the desired composition from H_2_O. The CN stretching vibration of the anions in the crystals is observed at 2072 cm^−1^, indicating the presence of terminal thio­cyanate anions (Fig. S1). Single crystal X-ray diffraction proves that the hitherto missing ansolvate has formed and XRPD investigations reveal the formation of a phase-pure sample (Fig. S2). Comparison of the experimental powder pattern obtained by solvent removal from the aceto­nitrile, ethanol and diethyl ether solvates with that calculated for the ansolvate proves that all of these crystalline phases are identical (Fig. 1 ▸). TG-DTA measurements show that the title compound decomposes in three steps, which are all accompanied by an endothermic event in the DTA curve (Fig. S3). The calculated mass loss per coligand amounts to 17.0%, which means that the first step (33.3%) is in reasonable agreement with the loss of two ligands and the second (15.7%) and third (14.9%) step with the loss of one ligand each, indicating the formation of additional compounds. 
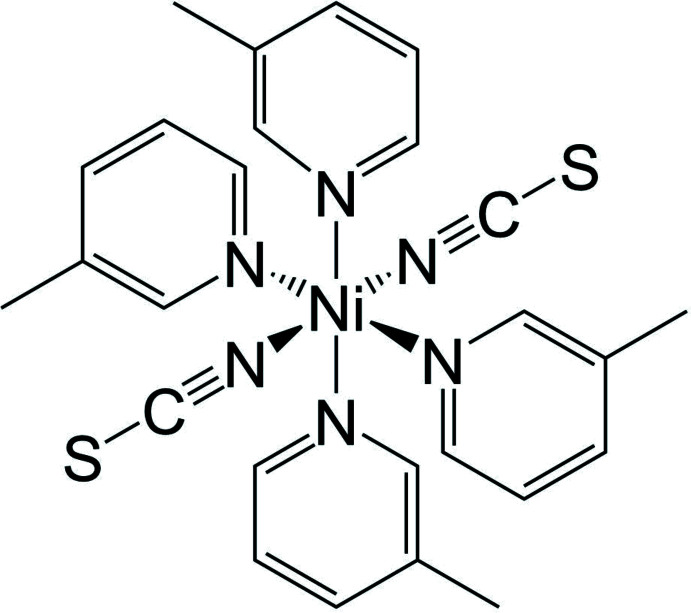


## 2. Structural commentary

**Figure 2:**
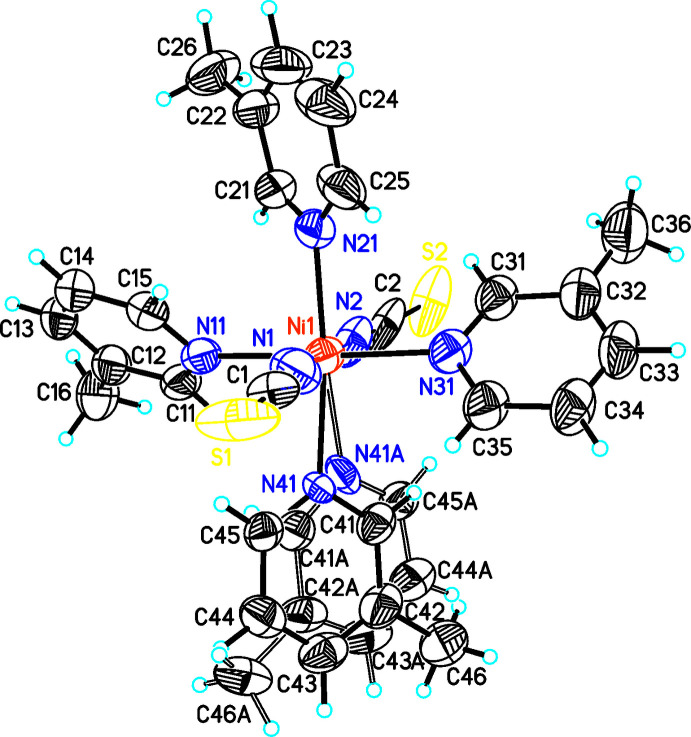
Crystal structure of the title compound with atom labeling and displacement ellipsoids drawn at the 50% probability level using *XP* in *SHELX*-*PC* (Sheldrick, 1996 ▸). The disorder of one of the 3-methyl­pyridine ligands is shown as full and open bonds.

The asymmetric unit of the title compound, Ni(NCS)_2_(3-methyl­pyridine)_4_, consists of one Ni^II^ cation, two thio­cyanate anions and four 3-methyl­pyridine coligands that occupy general positions. One of the 3-methyl­pyridine coligands is disordered and was refined using a split model (Fig. 2 ▸). In the crystal structure of the title compound, the nickel cations are sixfold coordinated by two terminal N-bonded thio­cyanate anions and four 3-methyl­pyridine coligands and from the bond lengths and angles it is obvious that the octa­hedra are slightly distorted (Table 1 ▸). This can also be seen from the octa­hedral angle variance (with a value of 11.2355°^2^) and the mean octa­hedral quadratic elongation (with a value of 1.0042) determined by the method of Robinson *et al.* (1971 ▸).

## 3. Supra­molecular features

**Figure 3:**
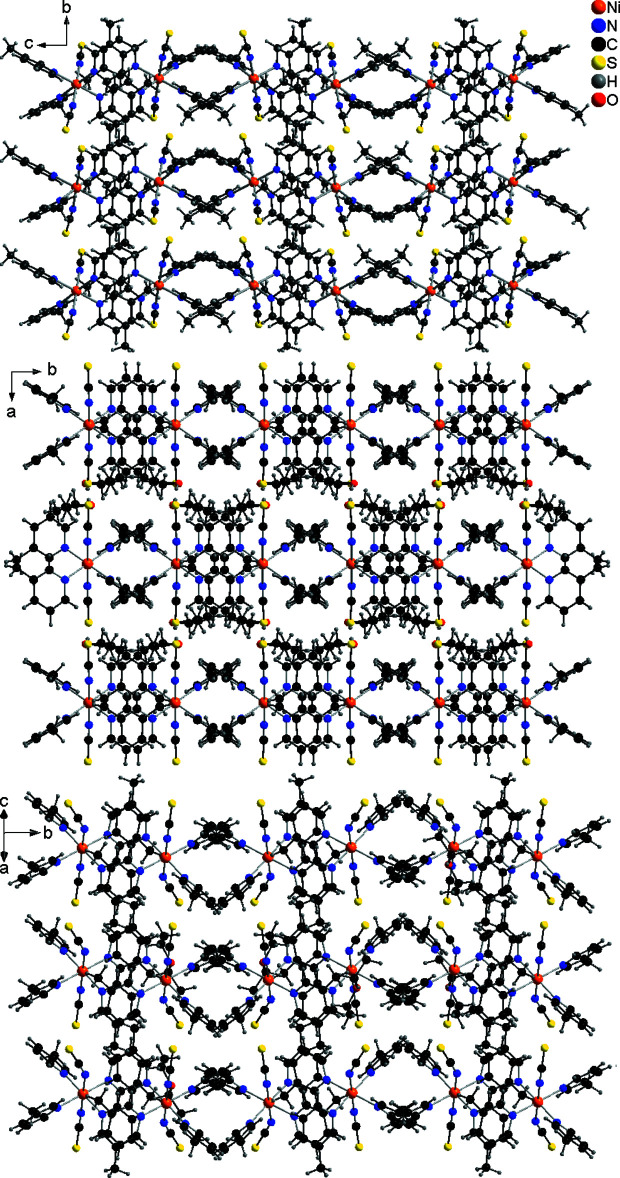
Crystal structure of the title compound drawn with *DIAMOND* (Brandenburg & Putz, 1999 ▸) with a view along the crystallographic *a*-axis (top) and of the compounds Ni(NCS)_2_(3-methyl­pyridine)_4_·2ethanol (mid) and Ni(NCS)_2_(3-methyl­pyridine)_4_·diethyl ether (bottom) retrieved from the literature (Krebs *et al.*, 2022 ▸).

In the crystal structure of the title compound, the discrete complexes are arranged into layers that are located in the *ab* plane (Fig. 3 ▸: top). These layers are separated from neighbouring layers by pairs of 3-methyl­pyridine ligands that show a butterfly-like arrangement. There are no indications for π–π stacking or inter­molecular hydrogen bonding. There are only C—H⋯N and C—H⋯S contacts, but from the distances and angles it is obvious that these are not significant inter­actions. The arrangement of the complexes in the title compound is similar to that in the solvates Ni(NCS)_2_(3-methyl­pyridine)_4_·ethanol and the isotypic compound Ni(NCS)_2_(3-methyl­pyridine)_4_·aceto­nitrile (Krebs *et al.*, 2022 ▸), indicating some structural relationship (Fig. 3 ▸). However, the third solvate, Ni(NCS)_2_(3-methyl­pyridine)_4_·diethyl ether (Krebs *et al.*, 2022 ▸) is not isotypic to the ethanol and aceto­nitrile solvates, yet also transforms into the title compound upon solvent removal. Even in this compound, a similar arrangement of the complexes is formed, which strongly suggests that the same crystalline ansolvate phase is particularly stable.

## 4. Database survey

Some compounds with 3-methyl­pyridine as coligand and transition-metal thio­cyanates other than Ni(NCS)_2_ (see *Chemical context*) were found in the CSD (version 5.43, last update November 2021; Groom *et al.*, 2016 ▸) using *ConQuest* (Bruno *et al.*, 2002 ▸). They include discrete complexes with Co(NCS)_2_ with an octa­hedral coordination around the metal center such as Co(NCS)_2_(3-methyl­pyridine)_4_ (EYAROM and EYAROM01; Boeckmann *et al.*, 2011 ▸ and Małecki *et al.*, 2012 ▸) and Co(NCS)_2_(3-methyl­pyridine)_2_(H_2_O)_2_ (EYAREC; Boeck­mann *et al.*, 2011 ▸) and a tedrahedral coordination as in Co(NCS)_2_(3-methyl­pyridine)_2_ (EYARIG; Boeckmann *et al.*, 2011 ▸). Some Cu(NCS)_2_ compounds are also known from the literature. These are the tetra­hedrally coordinated compound Cu(NCS)(3-methyl­pyridine)_2_ where thio­cyanate anions link the copper cations into chains (CUHBEM; Healy *et al.*, 1984 ▸), Cu(NCS)_2_(3-methyl­pyridine)_3_ with a fivefold trigonal–bipyramidal-like coordination (VEPBAT; Kabešová & Kožíšková, 1989 ▸), and Cu(NCS)_2_(3-methyl­pyridine)_2_ where the metal center is square planar and coordinated by two thio­cyanate anions and two 3-methyl­pyridine coligands (ABOTET; Handy *et al.*, 2017 ▸). Additionally, two isotypic iron and manganese complexes with the composition *M*(NCS)_2_(3-methyl­pyridine)_4_ (*M* = Fe, Mn) are reported (Ceglarska *et al.*, 2022 ▸). With Cd(NCS)_2_, only the octa­hedral complex Cd(NCS)_2_(3-methyl­pyridine)_2_ is known, in which the cadmium cations are bridged into chains by thio­cyanate anions (FIYGUP; Taniguchi *et al.*, 1987 ▸). There is also one zinc complex with the composition Zn(NCS)_2_(3-methyl­pyridine)_2_ (ETUSAO; Boeckmann & Näther, 2011 ▸), where the metal centers are tetra­hedrally coordinated. Finally, the two non-heterometallic complexes *catena*-[tetra­kis­(thio­cyanato)­bis­(3-methyl­pyridine)­mangan­ese­mercury] (NAQYOW; Małecki, 2017*a* ▸) and *catena*-[tetra­kis­(μ-thio­cyanato)­bis­(3-methyl­pyridine)­mercuryzinc (QAM­SIJ; Małecki, 2017*b* ▸) are also known.

## 5. Synthesis and crystallization

**Synthesis**

Ni(NCS)_2_ was purchased from Santa Cruz Biotechnology. 3-Methyl­pyridine (also known as 3-picoline) was purchased from Alfa Aesar.

Ni(NCS)_2_(3-methyl­pyridine)_4_: 0.25mmol Ni(SCN)_2_ (43.7 mg) and 2.5 mmol 3-methyl­pyridine (243 µl) where added to 1.5 mL deionized H_2_O and stored under hydro­thermal conditions for 2 d at 403 K. As a result, light-blue single crystals were obtained.

**Experimental details**

The data collection for single crystal structure analysis was performed using an XtaLAB Synergy, Dualflex, HyPix diffractometer from Rigaku with Cu *K*α radiation.

The XRPD measurements were performed with a Stoe Transmission Powder Diffraction System (STADI P) equipped with a MYTHEN 1K detector and a Johansson-type Ge(111) monochromator using Cu *K*α_1_ radiation (λ = 1.540598 Å).

The IR spectra were measured using an ATI Mattson Genesis Series FTIR Spectrometer, control software: *WINFIRST*, from ATI Mattson.

Thermogravimetry and differential thermoanalysis (TG-DTA) measurements were performed in a dynamic nitro­gen atmosphere in Al_2_O_3_ crucibles using a STA-PT 1000 thermobalance from Linseis. The instrument was calibrated using standard reference materials.

## 6. Refinement

All crystals are of poor quality and merohedrally twinned with at least two componenents that are difficult to separate as is obvious from a view along the b* direction (Fig. S4). Therefore, a twin refinement using data in HKLF-5 format was performed, leading to a BASF parameter of 0.457 (5). Refinement using anisotropic displacement parameters leads to relatively large components of the anisotropic displacement parameters, indicating static or dynamic disordering. For one of the four crystallographically independent 3-methyl­pyridine coligands, the disorder was resolved and this ligand was refined using a split model with restraints. The C-bound H atoms were positioned with idealized geometry (methyl H atoms allowed to rotate but not to tip) and were refined isotropically with *U*_iso_(H) = 1.5*U*_eq_(C) for methyl H atoms and with *U*_iso_(H) = 1.2 *U*_eq_(C) for all other H atoms using a riding model. Crystal data, data collection and structure refinement details are summarized in Table 2 ▸.

## Supplementary Material

Crystal structure: contains datablock(s) I. DOI: 10.1107/S2056989022011379/yz2025sup1.cif

Structure factors: contains datablock(s) I. DOI: 10.1107/S2056989022011379/yz2025Isup7.hkl

IR spectrum of the title compound. The value of the CN stretching vibration of the thiocyanate anions is given. DOI: 10.1107/S2056989022011379/yz2025sup3.png

Experimental (top) and calculated XRPD pattern (bottom) of the title compound. DOI: 10.1107/S2056989022011379/yz2025sup4.png

DTG (top) TG (mid) and DTA curve (bottom) of the title compound measured with 8C/min. The mass loss in % and the peak temperatures are given. DOI: 10.1107/S2056989022011379/yz2025sup5.png

View of the diffraction pattern of the crystal of the title compound along the b* direction. The two twin components are indicated in black and blue. DOI: 10.1107/S2056989022011379/yz2025sup6.png

CCDC reference: 2222139

Additional supporting information:  crystallographic information; 3D view; checkCIF report

## Figures and Tables

**Table 1:** Selected geometric parameters (Å, °)

Ni1—N1	2.064 (4)	Ni1—N31	2.126 (3)
Ni1—N2	2.037 (4)	Ni1—N41	2.193 (10)
Ni1—N11	2.124 (3)	Ni1—N41*A*	2.075 (11)
Ni1—N21	2.118 (3)		
			
N1—Ni1—N11	90.23 (15)	N2—Ni1—N41*A*	82.0 (3)
N1—Ni1—N21	90.76 (14)	N11—Ni1—N31	177.86 (12)
N1—Ni1—N31	90.57 (14)	N11—Ni1—N41	87.7 (3)
N1—Ni1—N41	83.7 (3)	N21—Ni1—N11	87.12 (12)
N1—Ni1—N41*A*	98.2 (3)	N21—Ni1—N31	90.89 (12)
N2—Ni1—N1	178.73 (15)	N21—Ni1—N41	172.4 (3)
N2—Ni1—N11	91.01 (14)	N31—Ni1—N41	94.4 (3)
N2—Ni1—N21	89.04 (14)	N41*A*—Ni1—N11	95.6 (3)
N2—Ni1—N31	88.17 (14)	N41*A*—Ni1—N21	170.6 (3)
N2—Ni1—N41	96.6 (3)	N41*A*—Ni1—N31	86.2 (3)

**Table 2:** Experimental details

Crystal data	
Chemical formula	[Ni(NCS)_2_(C_6_H_7_N)_4_]
*M*_r_	547.37
Crystal system, space group	Orthorhombic, *P* *b* *c* *a*
Temperature (K)	100
*a*, *b*, *c* (Å)	14.2012 (4), 15.2704 (4), 26.1738 (6)
*V* (Å^3^)	5676.0 (3)
*Z*	8
Radiation type	Cu *K*α
μ (mm^−1^)	2.55
Crystal size (mm)	0.15 × 0.1 × 0.1

Data collection	
Diffractometer	XtaLAB Synergy, Dualflex, HyPix
Absorption correction	Multi-scan (*CrysAlis PRO*; Rigaku OD, 2021 ▸)
*T*_min_, *T*_max_	0.814, 1.000
No. of measured, independent and observed [*I* > 2σ(*I*)] reflections	6767, 6767, 5975
*R*_int_	?
(sin θ/λ)_max_ (Å^−1^)	0.602

Refinement	
*R*[*F* ^2^ > 2σ(*F* ^2^)], *wR*(*F* ^2^), *S*	0.072, 0.203, 1.08
No. of reflections	6767
No. of parameters	386
No. of restraints	15
H-atom treatment	H-atom parameters constrained
Δρ_max_, Δρ_min_ (e Å^−3^)	0.74, −0.61

Computer programs: *CrysAlis PRO* (Rigaku OD, 2021 ▸), *SHELXT2014/5* (Sheldrick, 2015*a* ▸), *SHELXL2016/6* (Sheldrick, 2015*b* ▸), *DIAMOND* (Brandenburg & Putz, 1999 ▸) and *publCIF* (Westrip, 2010 ▸).

## supplementary crystallographic information

### Crystal data

**Table:**

[Ni(NCS)_2_(C_6_H_7_N)_4_]	*D*_x_ = 1.281 Mg m^−^^3^
*M**_r_* = 547.37	Cu *K*α radiation, λ = 1.54184 Å
Orthorhombic, *P**b**c**a*	Cell parameters from 11586 reflections
*a* = 14.2012 (4) Å	θ = 3.4–77.9°
*b* = 15.2704 (4) Å	µ = 2.55 mm^−^^1^
*c* = 26.1738 (6) Å	*T* = 100 K
*V* = 5676.0 (3) Å^3^	Block, light blue
*Z* = 8	0.15 × 0.1 × 0.1 mm
*F*(000) = 2288	

### Data collection

**Table:**

XtaLAB Synergy, Dualflex, HyPix diffractometer	6767 measured reflections
Radiation source: micro-focus sealed X-ray tube, PhotonJet (Cu) X-ray Source	6767 independent reflections
Mirror monochromator	5975 reflections with *I* > 2σ(*I*)
Detector resolution: 10.0000 pixels mm^-1^	θ_max_ = 68.3°, θ_min_ = 3.4°
ω scans	*h* = −15→17
Absorption correction: multi-scan (CrysAlisPro; Rigaku OD, 2021)	*k* = −17→18
*T*_min_ = 0.814, *T*_max_ = 1.000	*l* = −31→31

### Refinement

**Table:**

Refinement on *F*^2^	Primary atom site location: dual
Least-squares matrix: full	Hydrogen site location: inferred from neighbouring sites
*R*[*F*^2^ > 2σ(*F*^2^)] = 0.072	H-atom parameters constrained
*wR*(*F*^2^) = 0.203	*w* = 1/[σ^2^(*F*_o_^2^) + (0.0864*P*)^2^ + 8.6753*P*] where *P* = (*F*_o_^2^ + 2*F*_c_^2^)/3
*S* = 1.08	(Δ/σ)_max_ = 0.001
6767 reflections	Δρ_max_ = 0.74 e Å^−^^3^
386 parameters	Δρ_min_ = −0.61 e Å^−^^3^
15 restraints	

### Special details

**Table:**

Geometry. All esds (except the esd in the dihedral angle between two l.s. planes) are estimated using the full covariance matrix. The cell esds are taken into account individually in the estimation of esds in distances, angles and torsion angles; correlations between esds in cell parameters are only used when they are defined by crystal symmetry. An approximate (isotropic) treatment of cell esds is used for estimating esds involving l.s. planes.
Refinement. Refined as a two-component twin.

### Fractional atomic coordinates and isotropic or equivalent isotropic displacement parameters (Å^2^)

**Table:**

	*x*	*y*	*z*	*U*_iso_*/*U*_eq_	Occ. (<1)
Ni1	0.51000 (5)	0.76369 (4)	0.61618 (2)	0.0493 (2)	
N1	0.4176 (3)	0.8666 (3)	0.62644 (15)	0.0654 (9)	
C1	0.3581 (3)	0.9147 (3)	0.63485 (14)	0.0595 (10)	
S1	0.27254 (11)	0.98387 (12)	0.64613 (6)	0.0992 (6)	
N2	0.6035 (3)	0.6638 (2)	0.60680 (13)	0.0647 (10)	
C2	0.6745 (4)	0.6257 (3)	0.60497 (15)	0.0683 (14)	
S2	0.77540 (12)	0.57472 (10)	0.60158 (6)	0.0984 (6)	
N11	0.4125 (2)	0.7018 (2)	0.56639 (11)	0.0510 (8)	
C11	0.4102 (3)	0.6152 (3)	0.56289 (14)	0.0546 (10)	
H11	0.437953	0.582251	0.589725	0.065*	
C12	0.3696 (3)	0.5693 (3)	0.52235 (15)	0.0575 (10)	
C13	0.3294 (3)	0.6190 (3)	0.48387 (15)	0.0623 (11)	
H13	0.302533	0.591038	0.454903	0.075*	
C14	0.3283 (3)	0.7090 (3)	0.48761 (15)	0.0627 (11)	
H14	0.299610	0.743464	0.461734	0.075*	
C15	0.3698 (3)	0.7485 (3)	0.52963 (15)	0.0550 (9)	
H15	0.367899	0.810485	0.532513	0.066*	
C16	0.3721 (4)	0.4712 (3)	0.52121 (19)	0.0778 (14)	
H16A	0.422808	0.450120	0.543429	0.117*	
H16B	0.383374	0.451212	0.486156	0.117*	
H16C	0.311698	0.448037	0.533300	0.117*	
N21	0.5673 (2)	0.8250 (2)	0.55058 (12)	0.0503 (7)	
C21	0.5828 (3)	0.7784 (3)	0.50761 (15)	0.0570 (10)	
H21	0.577635	0.716422	0.509351	0.068*	
C22	0.6058 (3)	0.8161 (4)	0.46099 (17)	0.0750 (15)	
C23	0.6157 (4)	0.9059 (5)	0.4605 (3)	0.098 (2)	
H23	0.631190	0.934897	0.429489	0.117*	
C24	0.6034 (4)	0.9536 (4)	0.5041 (3)	0.0869 (17)	
H24	0.611759	1.015283	0.503588	0.104*	
C25	0.5787 (3)	0.9119 (3)	0.5489 (2)	0.0654 (12)	
H25	0.569678	0.945459	0.579052	0.079*	
C26	0.6213 (4)	0.7585 (5)	0.41446 (19)	0.102 (2)	
H26A	0.673140	0.717732	0.421148	0.153*	
H26B	0.636960	0.795217	0.384962	0.153*	
H26C	0.563707	0.725337	0.407245	0.153*	
N31	0.6110 (3)	0.8264 (2)	0.66364 (12)	0.0526 (8)	
C31	0.7026 (3)	0.8279 (3)	0.65102 (15)	0.0560 (9)	
H31	0.720195	0.803220	0.619100	0.067*	
C32	0.7734 (3)	0.8631 (3)	0.68143 (17)	0.0636 (11)	
C33	0.7455 (4)	0.8989 (3)	0.72751 (18)	0.0705 (12)	
H33	0.790971	0.923652	0.749867	0.085*	
C34	0.6519 (4)	0.8988 (3)	0.74102 (17)	0.0686 (12)	
H34	0.632489	0.923237	0.772691	0.082*	
C35	0.5854 (3)	0.8623 (3)	0.70773 (16)	0.0602 (10)	
H35	0.520662	0.863337	0.716855	0.072*	
C36	0.8747 (4)	0.8623 (5)	0.6636 (2)	0.0935 (18)	
H36A	0.884808	0.812250	0.640852	0.140*	
H36B	0.916506	0.857366	0.693291	0.140*	
H36C	0.888383	0.916723	0.645267	0.140*	
N41	0.4324 (8)	0.7054 (7)	0.6801 (3)	0.038 (2)	0.508 (9)
C41	0.4817 (8)	0.6676 (6)	0.7171 (3)	0.047 (2)	0.508 (9)
H41	0.548178	0.663650	0.713665	0.056*	0.508 (9)
C42	0.4391 (10)	0.6336 (8)	0.7607 (5)	0.060 (4)	0.508 (9)
C43	0.3410 (8)	0.6417 (6)	0.7648 (4)	0.058 (3)	0.508 (9)
H43	0.309505	0.619128	0.794012	0.070*	0.508 (9)
C44	0.2899 (8)	0.6821 (6)	0.7267 (3)	0.055 (2)	0.508 (9)
H44	0.223478	0.688128	0.729071	0.066*	0.508 (9)
C45	0.3393 (8)	0.7135 (6)	0.6849 (3)	0.044 (2)	0.508 (9)
H45	0.305316	0.742056	0.658496	0.053*	0.508 (9)
C46	0.4967 (7)	0.5905 (8)	0.8022 (4)	0.077 (3)	0.508 (9)
H46A	0.553891	0.565381	0.787301	0.115*	0.508 (9)
H46B	0.459517	0.544001	0.818299	0.115*	0.508 (9)
H46C	0.513938	0.634263	0.827964	0.115*	0.508 (9)
N41A	0.4738 (7)	0.6913 (7)	0.6803 (4)	0.045 (3)	0.492 (9)
C41A	0.3824 (10)	0.6897 (7)	0.6936 (4)	0.044 (2)	0.492 (9)
H41A	0.339204	0.717790	0.671248	0.053*	0.492 (9)
C42A	0.3452 (8)	0.6500 (6)	0.7378 (4)	0.052 (3)	0.492 (9)
C43A	0.4107 (10)	0.6097 (8)	0.7696 (5)	0.060 (4)	0.492 (9)
H43A	0.390213	0.583224	0.800541	0.072*	0.492 (9)
C44A	0.5050 (8)	0.6076 (7)	0.7569 (3)	0.061 (2)	0.492 (9)
H44A	0.549538	0.579352	0.778394	0.073*	0.492 (9)
C45A	0.5335 (8)	0.6479 (6)	0.7114 (3)	0.048 (2)	0.492 (9)
H45A	0.598007	0.644640	0.702042	0.058*	0.492 (9)
C46A	0.2399 (7)	0.6515 (7)	0.7488 (4)	0.067 (3)	0.492 (9)
H46D	0.211614	0.703204	0.732851	0.101*	0.492 (9)
H46E	0.229632	0.653656	0.785833	0.101*	0.492 (9)
H46F	0.210660	0.598466	0.734885	0.101*	0.492 (9)

### Atomic displacement parameters (Å^2^)

**Table:**

	*U*^11^	*U*^22^	*U*^33^	*U*^12^	*U*^13^	*U*^23^
Ni1	0.0694 (5)	0.0412 (4)	0.0372 (4)	−0.0156 (3)	−0.0019 (3)	−0.0012 (2)
N1	0.060 (2)	0.070 (2)	0.066 (2)	−0.0123 (19)	0.0107 (17)	−0.0036 (19)
C1	0.066 (3)	0.071 (3)	0.0416 (19)	−0.008 (2)	0.0102 (17)	0.0098 (18)
S1	0.1028 (10)	0.1118 (12)	0.0831 (9)	0.0356 (9)	0.0467 (8)	0.0492 (8)
N2	0.098 (3)	0.0470 (19)	0.0487 (18)	−0.003 (2)	−0.0278 (18)	−0.0025 (15)
C2	0.119 (4)	0.041 (2)	0.045 (2)	0.004 (3)	−0.040 (2)	−0.0070 (17)
S2	0.1265 (12)	0.0774 (9)	0.0913 (9)	0.0348 (8)	−0.0594 (9)	−0.0317 (7)
N11	0.0614 (19)	0.0553 (19)	0.0365 (14)	−0.0197 (15)	0.0011 (12)	0.0000 (13)
C11	0.069 (2)	0.058 (2)	0.0371 (17)	−0.0218 (19)	0.0012 (16)	−0.0019 (16)
C12	0.059 (2)	0.066 (3)	0.0469 (19)	−0.019 (2)	0.0017 (16)	−0.0111 (18)
C13	0.058 (2)	0.082 (3)	0.047 (2)	−0.022 (2)	−0.0059 (17)	−0.010 (2)
C14	0.054 (2)	0.084 (3)	0.049 (2)	−0.015 (2)	−0.0061 (17)	0.005 (2)
C15	0.052 (2)	0.064 (2)	0.049 (2)	−0.0147 (19)	0.0028 (16)	0.0041 (18)
C16	0.090 (3)	0.075 (3)	0.069 (3)	−0.020 (3)	−0.012 (2)	−0.024 (2)
N21	0.0485 (16)	0.0506 (18)	0.0518 (17)	−0.0086 (14)	−0.0036 (13)	0.0102 (14)
C21	0.053 (2)	0.072 (3)	0.046 (2)	−0.0062 (19)	−0.0031 (16)	0.0132 (19)
C22	0.052 (2)	0.118 (5)	0.055 (2)	−0.007 (3)	0.0010 (18)	0.031 (3)
C23	0.063 (3)	0.132 (6)	0.098 (4)	−0.004 (3)	0.008 (3)	0.073 (4)
C24	0.065 (3)	0.076 (3)	0.121 (5)	−0.005 (3)	0.002 (3)	0.048 (3)
C25	0.050 (2)	0.058 (3)	0.088 (3)	−0.0111 (19)	−0.003 (2)	0.025 (2)
C26	0.090 (4)	0.169 (7)	0.047 (3)	−0.003 (4)	0.008 (2)	0.021 (3)
N31	0.074 (2)	0.0369 (16)	0.0472 (16)	−0.0094 (15)	−0.0043 (15)	−0.0048 (13)
C31	0.074 (3)	0.045 (2)	0.049 (2)	−0.0072 (19)	−0.0067 (18)	−0.0036 (16)
C32	0.068 (3)	0.058 (3)	0.065 (2)	−0.004 (2)	−0.017 (2)	−0.003 (2)
C33	0.083 (3)	0.060 (3)	0.068 (3)	−0.011 (2)	−0.021 (2)	−0.016 (2)
C34	0.092 (3)	0.054 (2)	0.060 (2)	−0.007 (2)	−0.012 (2)	−0.020 (2)
C35	0.080 (3)	0.047 (2)	0.054 (2)	−0.010 (2)	−0.0031 (19)	−0.0122 (17)
C36	0.075 (3)	0.118 (5)	0.087 (4)	−0.002 (3)	−0.018 (3)	−0.014 (4)
N41	0.031 (6)	0.052 (5)	0.032 (3)	−0.015 (5)	0.006 (4)	−0.004 (3)
C41	0.059 (6)	0.039 (4)	0.042 (5)	−0.001 (4)	−0.008 (4)	0.001 (3)
C42	0.086 (10)	0.044 (6)	0.050 (7)	−0.016 (6)	−0.015 (6)	0.014 (4)
C43	0.087 (8)	0.044 (5)	0.044 (5)	−0.013 (5)	0.006 (5)	0.000 (4)
C44	0.052 (5)	0.053 (5)	0.060 (5)	−0.008 (4)	0.008 (4)	−0.001 (4)
C45	0.056 (6)	0.040 (5)	0.037 (4)	−0.010 (4)	−0.002 (4)	−0.005 (3)
C46	0.083 (7)	0.086 (7)	0.062 (6)	−0.008 (5)	−0.012 (4)	0.026 (5)
N41A	0.033 (5)	0.050 (5)	0.051 (5)	−0.017 (4)	0.007 (5)	−0.016 (4)
C41A	0.049 (8)	0.044 (5)	0.039 (5)	−0.002 (5)	0.001 (5)	−0.008 (4)
C42A	0.074 (7)	0.041 (5)	0.042 (5)	−0.012 (5)	0.019 (6)	−0.012 (4)
C43A	0.092 (10)	0.044 (7)	0.043 (5)	−0.005 (6)	0.021 (5)	−0.001 (5)
C44A	0.079 (7)	0.057 (6)	0.047 (5)	0.001 (5)	−0.002 (4)	0.005 (4)
C45A	0.055 (6)	0.050 (5)	0.040 (4)	−0.003 (4)	0.002 (4)	−0.004 (3)
C46A	0.069 (6)	0.065 (6)	0.067 (5)	−0.005 (5)	0.031 (5)	0.000 (5)

### Geometric parameters (Å, º)

**Table:**

Ni1—N1	2.064 (4)	C31—H31	0.9500
Ni1—N2	2.037 (4)	C31—C32	1.391 (6)
Ni1—N11	2.124 (3)	C32—C33	1.383 (7)
Ni1—N21	2.118 (3)	C32—C36	1.511 (7)
Ni1—N31	2.126 (3)	C33—H33	0.9500
Ni1—N41	2.193 (10)	C33—C34	1.375 (7)
Ni1—N41A	2.075 (11)	C34—H34	0.9500
N1—C1	1.142 (6)	C34—C35	1.400 (6)
C1—S1	1.637 (5)	C35—H35	0.9500
N2—C2	1.165 (6)	C36—H36A	0.9800
C2—S2	1.633 (6)	C36—H36B	0.9800
N11—C11	1.326 (5)	C36—H36C	0.9800
N11—C15	1.342 (5)	N41—C41	1.326 (12)
C11—H11	0.9500	N41—C45	1.333 (11)
C11—C12	1.396 (5)	C41—H41	0.9500
C12—C13	1.384 (6)	C41—C42	1.394 (14)
C12—C16	1.499 (7)	C42—C43	1.403 (14)
C13—H13	0.9500	C42—C46	1.510 (13)
C13—C14	1.378 (7)	C43—H43	0.9500
C14—H14	0.9500	C43—C44	1.378 (13)
C14—C15	1.386 (6)	C44—H44	0.9500
C15—H15	0.9500	C44—C45	1.385 (12)
C16—H16A	0.9800	C45—H45	0.9500
C16—H16B	0.9800	C46—H46A	0.9800
C16—H16C	0.9800	C46—H46B	0.9800
N21—C21	1.349 (5)	C46—H46C	0.9800
N21—C25	1.337 (6)	N41A—C41A	1.343 (12)
C21—H21	0.9500	N41A—C45A	1.349 (13)
C21—C22	1.388 (6)	C41A—H41A	0.9500
C22—C23	1.379 (9)	C41A—C42A	1.410 (14)
C22—C26	1.518 (9)	C42A—C43A	1.392 (15)
C23—H23	0.9500	C42A—C46A	1.524 (13)
C23—C24	1.365 (10)	C43A—H43A	0.9500
C24—H24	0.9500	C43A—C44A	1.381 (14)
C24—C25	1.380 (7)	C44A—H44A	0.9500
C25—H25	0.9500	C44A—C45A	1.398 (11)
C26—H26A	0.9800	C45A—H45A	0.9500
C26—H26B	0.9800	C46A—H46D	0.9800
C26—H26C	0.9800	C46A—H46E	0.9800
N31—C31	1.342 (6)	C46A—H46F	0.9800
N31—C35	1.329 (5)		
			
N1—Ni1—N11	90.23 (15)	C31—N31—Ni1	121.2 (3)
N1—Ni1—N21	90.76 (14)	C35—N31—Ni1	120.6 (3)
N1—Ni1—N31	90.57 (14)	C35—N31—C31	118.2 (4)
N1—Ni1—N41	83.7 (3)	N31—C31—H31	117.7
N1—Ni1—N41A	98.2 (3)	N31—C31—C32	124.5 (4)
N2—Ni1—N1	178.73 (15)	C32—C31—H31	117.7
N2—Ni1—N11	91.01 (14)	C31—C32—C36	120.6 (4)
N2—Ni1—N21	89.04 (14)	C33—C32—C31	116.4 (4)
N2—Ni1—N31	88.17 (14)	C33—C32—C36	123.0 (4)
N2—Ni1—N41	96.6 (3)	C32—C33—H33	120.0
N2—Ni1—N41A	82.0 (3)	C34—C33—C32	120.1 (4)
N11—Ni1—N31	177.86 (12)	C34—C33—H33	120.0
N11—Ni1—N41	87.7 (3)	C33—C34—H34	120.3
N21—Ni1—N11	87.12 (12)	C33—C34—C35	119.5 (4)
N21—Ni1—N31	90.89 (12)	C35—C34—H34	120.3
N21—Ni1—N41	172.4 (3)	N31—C35—C34	121.3 (5)
N31—Ni1—N41	94.4 (3)	N31—C35—H35	119.3
N41A—Ni1—N11	95.6 (3)	C34—C35—H35	119.3
N41A—Ni1—N21	170.6 (3)	C32—C36—H36A	109.5
N41A—Ni1—N31	86.2 (3)	C32—C36—H36B	109.5
C1—N1—Ni1	170.3 (4)	C32—C36—H36C	109.5
N1—C1—S1	179.3 (4)	H36A—C36—H36B	109.5
C2—N2—Ni1	160.5 (3)	H36A—C36—H36C	109.5
N2—C2—S2	178.4 (5)	H36B—C36—H36C	109.5
C11—N11—Ni1	120.2 (3)	C41—N41—Ni1	117.8 (7)
C11—N11—C15	118.0 (3)	C41—N41—C45	119.7 (9)
C15—N11—Ni1	119.9 (3)	C45—N41—Ni1	122.2 (6)
N11—C11—H11	117.8	N41—C41—H41	119.0
N11—C11—C12	124.3 (4)	N41—C41—C42	122.0 (10)
C12—C11—H11	117.8	C42—C41—H41	119.0
C11—C12—C16	120.5 (4)	C41—C42—C43	117.4 (9)
C13—C12—C11	116.6 (4)	C41—C42—C46	121.1 (11)
C13—C12—C16	122.9 (4)	C43—C42—C46	121.4 (10)
C12—C13—H13	120.0	C42—C43—H43	119.8
C14—C13—C12	120.0 (4)	C44—C43—C42	120.5 (10)
C14—C13—H13	120.0	C44—C43—H43	119.8
C13—C14—H14	120.5	C43—C44—H44	121.3
C13—C14—C15	119.1 (4)	C43—C44—C45	117.4 (11)
C15—C14—H14	120.5	C45—C44—H44	121.3
N11—C15—C14	122.0 (4)	N41—C45—C44	123.0 (10)
N11—C15—H15	119.0	N41—C45—H45	118.5
C14—C15—H15	119.0	C44—C45—H45	118.5
C12—C16—H16A	109.5	C42—C46—H46A	109.5
C12—C16—H16B	109.5	C42—C46—H46B	109.5
C12—C16—H16C	109.5	C42—C46—H46C	109.5
H16A—C16—H16B	109.5	H46A—C46—H46B	109.5
H16A—C16—H16C	109.5	H46A—C46—H46C	109.5
H16B—C16—H16C	109.5	H46B—C46—H46C	109.5
C21—N21—Ni1	120.3 (3)	C41A—N41A—Ni1	117.3 (7)
C25—N21—Ni1	120.8 (3)	C41A—N41A—C45A	116.2 (10)
C25—N21—C21	118.4 (4)	C45A—N41A—Ni1	126.5 (7)
N21—C21—H21	118.2	N41A—C41A—H41A	117.2
N21—C21—C22	123.5 (5)	N41A—C41A—C42A	125.6 (11)
C22—C21—H21	118.2	C42A—C41A—H41A	117.2
C21—C22—C26	119.9 (5)	C41A—C42A—C46A	121.1 (12)
C23—C22—C21	116.4 (5)	C43A—C42A—C41A	115.6 (10)
C23—C22—C26	123.6 (5)	C43A—C42A—C46A	123.3 (10)
C22—C23—H23	119.7	C42A—C43A—H43A	119.6
C24—C23—C22	120.6 (5)	C44A—C43A—C42A	120.9 (10)
C24—C23—H23	119.7	C44A—C43A—H43A	119.6
C23—C24—H24	120.1	C43A—C44A—H44A	120.8
C23—C24—C25	119.8 (5)	C43A—C44A—C45A	118.4 (10)
C25—C24—H24	120.1	C45A—C44A—H44A	120.8
N21—C25—C24	121.1 (5)	N41A—C45A—C44A	123.2 (10)
N21—C25—H25	119.4	N41A—C45A—H45A	118.4
C24—C25—H25	119.4	C44A—C45A—H45A	118.4
C22—C26—H26A	109.5	C42A—C46A—H46D	109.5
C22—C26—H26B	109.5	C42A—C46A—H46E	109.5
C22—C26—H26C	109.5	C42A—C46A—H46F	109.5
H26A—C26—H26B	109.5	H46D—C46A—H46E	109.5
H26A—C26—H26C	109.5	H46D—C46A—H46F	109.5
H26B—C26—H26C	109.5	H46E—C46A—H46F	109.5
