# Supplementary figures and images for: Synthesis, crystal structure and thermal properties of tetra­kis­(3-methyl­pyridine-κN)bis­(thio­cyanato-κN)nickel(II)
Source: Acta Crystallogr E Crystallogr Commun. 2023 Jan 1;79(Pt 1):19–23. doi: 10.1107/S2056989022011379 (PMC9815138; doi:10.1107/S2056989022011379)

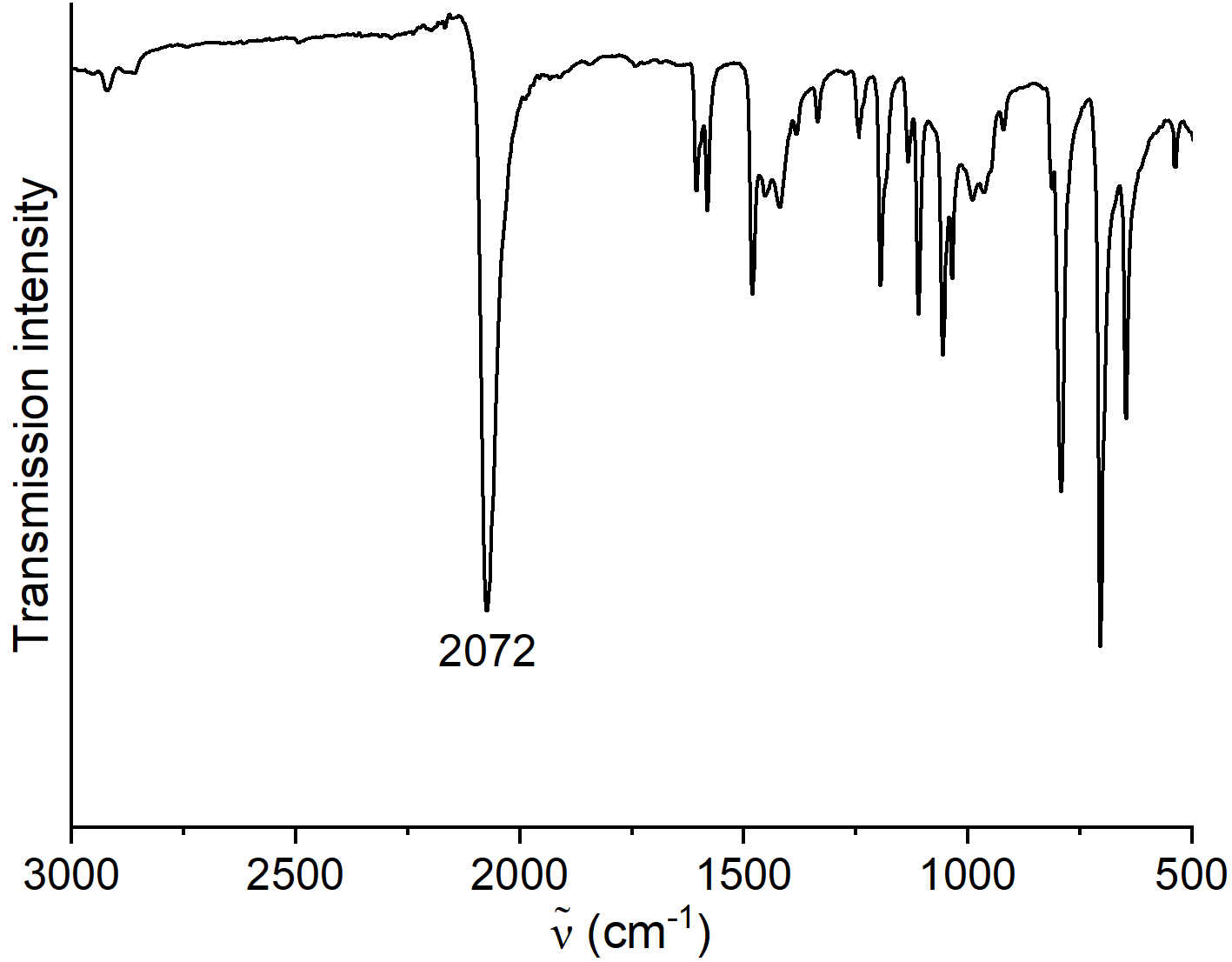

Supplement: Supplementary file 3 [file e-79-00019-sup3.png]

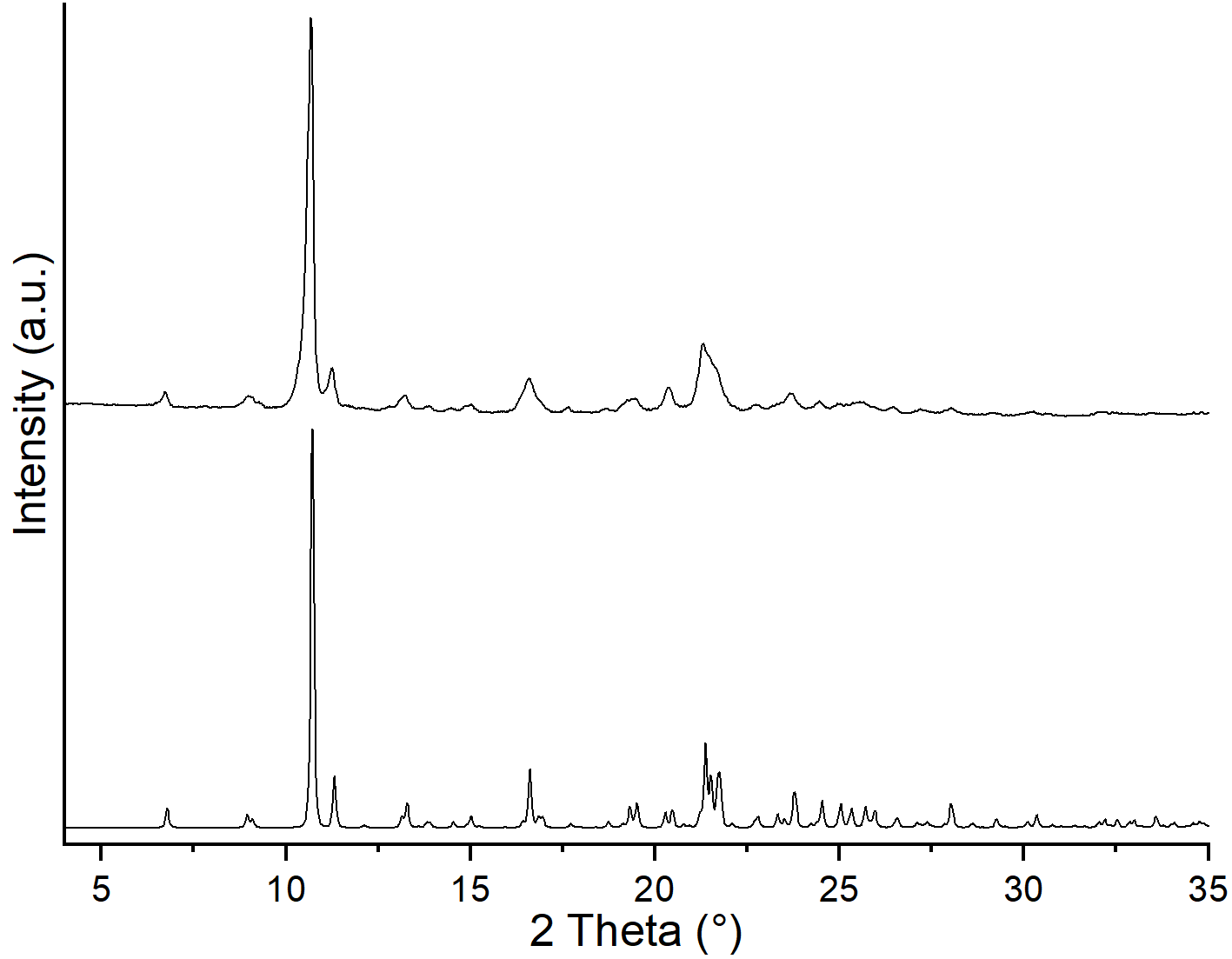

Supplement: Supplementary file 4 [file e-79-00019-sup4.png]

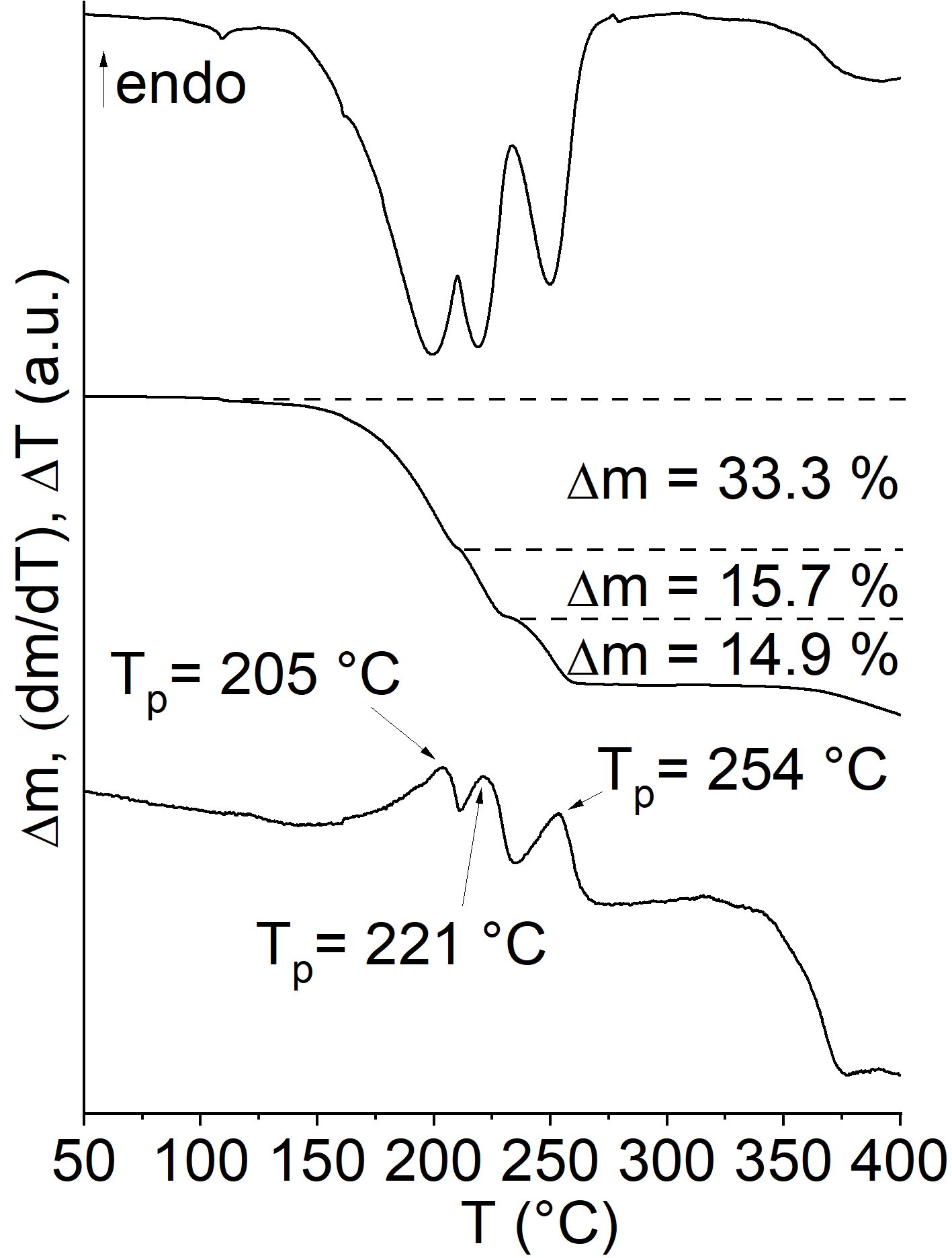

Supplement: Supplementary file 5 [file e-79-00019-sup5.png]

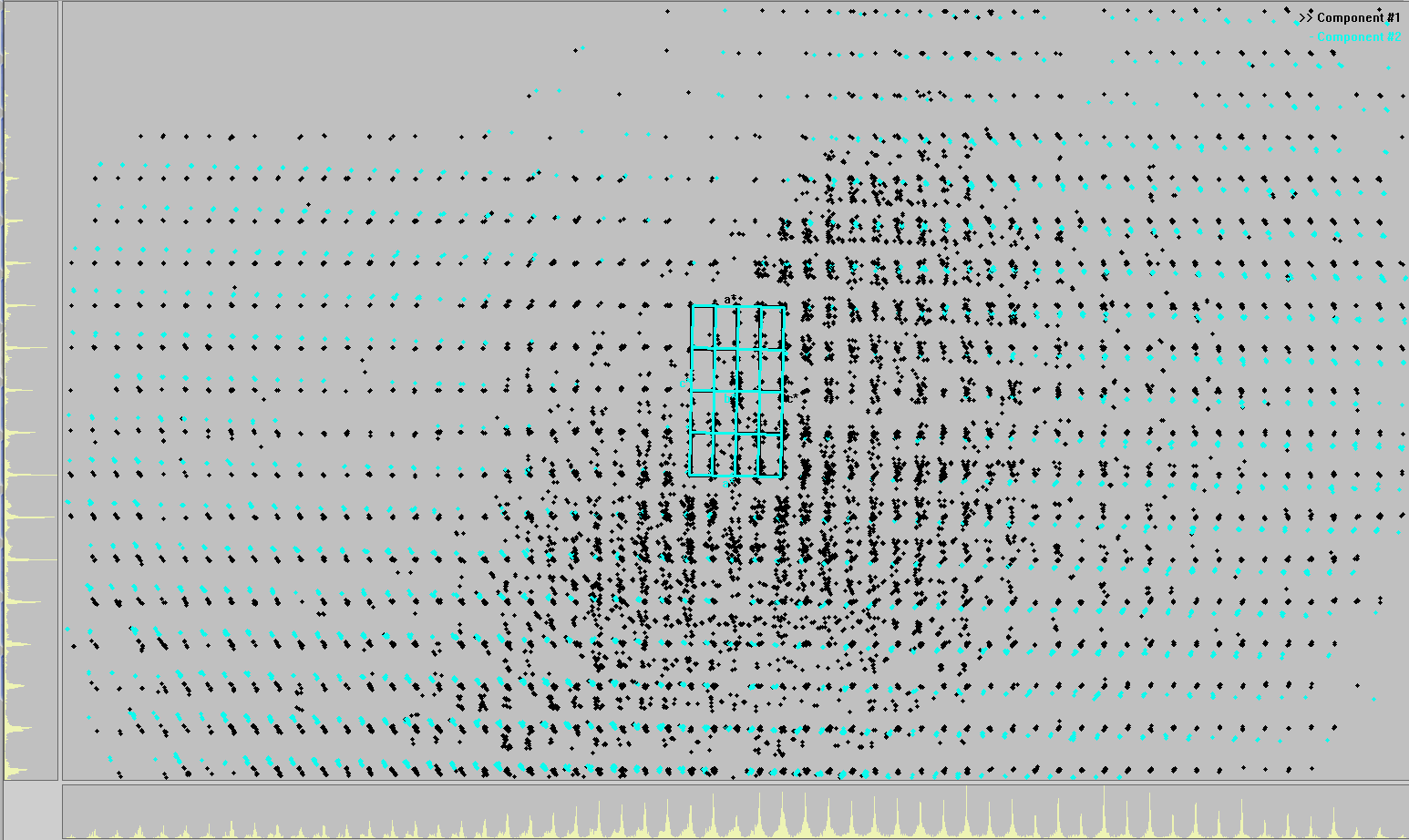

Supplement: Supplementary file 6 [file e-79-00019-sup6.png]
